# Physical activity and anxiety during the COVID-19 pandemic in Tanzania: insights for public health policy in low-income contexts

**DOI:** 10.3389/fpubh.2024.1483153

**Published:** 2024-12-23

**Authors:** Joyce Sifa Ndabi, Alfa Simwanza, JohnBosco C. Chukwuorji, Dawn Tladi, Rosemary C. Muomah, Sampson K. Nwonyi, Doris Akosua Tay, Dale Joachim, Leapetswe Malete, Clement Adamba, Vida Korleki Nyawornota, Oscar Nyanyo Nyanynofio, Samuel Kofi Donkor, Reginald Ocansey

**Affiliations:** ^1^Department of Physical Education and Sports Sciences, University of Dares Salaam, Dares Salaam, Tanzania; ^2^Department of Psychology, University of Nigeria, Nsukka, Nigeria; ^3^CS Mott Department of Public Health, Michigan State University, Michigan, MI, United States; ^4^Department of Sport Science, University of Botswana, Gaborone, Botswana; ^5^Department of Psychological Medicine, University of Nigeria, Enugu Campus, Nigeria; ^6^Department of Psychology, Ebonyi State University, Abakaliki, Nigeria; ^7^Department of Physical Education and Sport Studies, University of Ghana, Accra, Ghana; ^8^Speech Science, Sonde Health, Boston, MA, United States; ^9^Department of Kinesiology, Michigan State University, East Lansing, Michigan, MI, United States; ^10^School of Education and Leadership Studies, University of Ghana, Accra, Ghana; ^11^School of Sports and Exercise Medicine, University of Health and Allied Sciences, Ho, Ghana; ^12^Department of Health, Physical Education, Recreation, and Sports, University of Education, Winneba, Ghana

**Keywords:** physical activity, anxiety, COVID-19 pandemic, Tanzania, public health, policy, low-income contexts

## Abstract

**Background:**

The COVID-19 pandemic heightened anxiety levels globally, disproportionately affecting low-and middle-income countries (LMICs). Physical activity (PA) has shown potential to alleviate mental health challenges, including anxiety. This study explores the relationship between PA and anxiety among Tanzanian adults during the pandemic, examining whether self-reported health status moderates this relationship and identifying demographic variations.

**Methods:**

Data from 213 adults were collected using the International Physical Activity Questionnaire-Short Form (IPAQ-SF) and Generalized Anxiety Disorder-7 (GAD-7) scale. Self-reported health was also reported.

**Results:**

Vigorous physical activity was significantly associated with lower anxiety, while moderate physical activity showed weaker effects. Walking and sedentary behavior were not significantly associated with anxiety. Health status did not moderate these relationships, but subgroup analyses indicated stronger effects of vigorous physical activity among men and younger adults.

**Conclusion:**

These findings highlight the relevance of PA, particularly vigorous and moderate physical activity, in supporting mental health in LMIC contexts such as Tanzania. The findings further underscore the importance of targeted, culturally relevant physical activity interventions in LMICs to mitigate anxiety and enhance mental health resilience.

## 1 Introduction

### 1.1 Background

Physical activity (PA) is a well-established determinant of mental health, providing significant benefits in alleviating symptoms of anxiety and depression. The psychological benefits of PA are mediated by physiological mechanisms, including the regulation of stress hormones, such as cortisol, and the stimulation of endorphin release, which fosters a sense of well-being. In addition, PA enhances self-efficacy and provides a distraction from stressors, contributing to improved mental resilience.

Despite the extensive research on PA and mental health, most studies have been conducted in high-income countries (HICs). These studies often emphasize structured PA, such as gym workouts or leisure-time exercise, which are facilitated by ample resources and recreational facilities. In low-and middle-income countries (LMICs), such as Tanzania, the context is markedly different. PA is frequently incidental, arising from occupational tasks, transportation, or household chores, rather than deliberate health-promoting activities. Moreover, cultural norms, socio-economic constraints, and limited infrastructure often restrict access to recreational PA opportunities. This disparity underscores the need for context-specific research to explore how PA influences mental health in LMICs, particularly during periods of crisis.

### 1.2 Study context

The COVID-19 pandemic created unprecedented global disruptions, with mental health challenges intensifying due to uncertainties surrounding health, economic instability, and social isolation. In HICs, strict lockdown measures significantly reduced opportunities for PA, exacerbating mental health issues. Tanzania, however, adopted a distinct approach, imposing minimal lockdown measures to preserve economic activity. This strategy provided a unique setting to investigate the relationship between PA and anxiety during the pandemic, as individuals in Tanzania maintained relatively normal mobility patterns.

Understanding the nuances of PA’s impact on mental health in Tanzania can inform public health strategies that address the specific challenges and opportunities in LMICs. By focusing on the Tanzanian context, this study aims to provide actionable insights into how PA can be leveraged to mitigate anxiety during crises.

### 1.3 Study objectives

The primary objectives of this study were to examine the relationship between different intensities of PA—vigorous, moderate, and walking—and anxiety levels among Tanzanian adults. Additionally, the study aimed to investigate whether self-reported health status moderated these relationships and to explore demographic variations, including gender and age, in the mental health benefits of PA. These objectives seek to contribute to the global evidence base on PA and mental health, with a focus on the unique socio-economic and cultural contexts of LMICs.

### 1.4 Research questions

1. To examine the relationship between different levels of physical activity (vigorous, moderate, walking, and sedentary behavior) and anxiety among the Tanzanian population during the COVID-19 pandemic.
2. To investigate whether self-reported health status moderates the relationship between physical activity and anxiety.
3. To explore subgroup differences in the physical activity-anxiety relationship based on demographic factors such as gender and age.
4. To provide evidence-based insights for developing culturally and socio-economically tailored public health interventions that leverage physical activity to address anxiety in low-resource settings.

### 1.5 Research hypotheses

#### 1.5.1 Primary hypothesis

Higher levels of physical activity (vigorous and moderate) will be significantly associated with lower anxiety scores, while walking and sedentary behavior will not exhibit significant associations with anxiety.

#### 1.5.2 Moderation hypothesis

Self-reported health status will moderate the relationship between physical activity and anxiety, with stronger associations observed among individuals reporting better health.

#### 1.5.3 Subgroup hypothesis

The relationship between physical activity and anxiety will vary by demographic subgroups, with greater benefits of vigorous and moderate physical activity observed among men and younger adults.

## 2 Literature review

Physical activity (PA) is widely recognized for its myriad benefits on physical and mental health (1). Regular engagement in PA has been linked to reduced risks of chronic diseases, improved cardiovascular health, enhanced musculoskeletal strength, and better mental health outcomes (2). The mental health benefits of PA include reductions in symptoms of anxiety, depression, and stress, as well as improvements in mood and overall well-being (3). The International Physical Activity Questionnaire (IPAQ) is a standardized self-report measure developed to facilitate cross-national monitoring of PA and inactivity. The IPAQ exists in two versions: the short form (IPAQ-SF) and the long form (IPAQ-LF). The IPAQ-SF, utilized in this study, consists of seven items that assess the frequency and duration of vigorous and moderate physical activity, walking, and sitting over the last 7 days (4).

Anxiety is a prevalent mental health condition characterized by excessive worry, fear, and physical symptoms such as increased heart rate and muscle tension. It can significantly impair daily functioning and quality of life (5). The COVID-19 pandemic has exacerbated anxiety levels globally due to uncertainties, health threats, and social isolation measures (6). The Generalized Anxiety Disorder 7-item (GAD-7) scale is a brief self-report instrument used to screen for and assess the severity of generalized anxiety disorder.

PA emerged as a critical coping mechanism in high-income countries (HICs), where studies extensively documented its protective effects. However, there is limited evidence on the PA-anxiety relationship in low-and middle-income countries (LMICs), such as Tanzania, where socio-economic and cultural contexts differ significantly from HICs (7, 8). This study contributes to filling this gap by examining the role of PA in alleviating anxiety during the pandemic and exploring demographic and health-related moderators of this relationship.

Research consistently shows that vigorous and moderate physical activity can reduce anxiety symptoms through physiological mechanisms, such as the regulation of cortisol levels and the release of endorphins, and psychological mechanisms, including enhanced self-efficacy and stress resilience (3, 9). Studies in Sub-Saharan Africa emphasize the importance of understanding PA behaviors in contexts where exercise is often integrated into daily life, such as through occupational or transportation-related activities, rather than being a structured health-promoting behavior (10), highlight the additional challenges imposed by the pandemic, including restricted access to exercise spaces and heightened economic pressures, which disproportionately affected populations in LMICs.

In Tanzania, where economic disparities and cultural norms shape PA engagement, structured PA is not always accessible or prioritized. For example, women often face societal expectations that limit their ability to participate in recreational exercise (11). Moreover, walking, a common form of PA in Tanzania, is frequently utilitarian rather than recreational, and thus may not provide the same mental health benefits as deliberate, high-intensity PA (12). Previous studies underscore the importance of distinguishing between different PA types and their mental health impacts, particularly in LMICs, where daily activities are often dictated by necessity rather than leisure (7, 13).

The Tanzanian response to the COVID-19 pandemic offers a unique context for exploring the PA-anxiety relationship (12). Unlike countries with strict lockdowns, Tanzania maintained relative mobility during the pandemic, yet the economic and social stresses likely heightened anxiety levels. Understanding how various PA intensities—vigorous, moderate, and walking—relate to anxiety within this setting is crucial for informing public health interventions. Recent studies suggest that vigorous physical activity may offer the greatest mental health benefits, with moderate physical activity showing weaker but still significant effects (9, 10). However, the potential role of health status as a moderator of these relationships remains underexplored, particularly in LMICs.

This study addresses these gaps by examining the PA-anxiety relationship among Tanzanian adults, exploring whether self-reported health status moderates these effects, and identifying demographic differences in PA benefits. Findings from this research will provide valuable insights into culturally and contextually appropriate strategies for promoting PA to improve mental health in Tanzania and similar LMICs.

## 3 Methods

### 3.1 Study design

This study utilized a cross-sectional design to investigate the relationship between physical activity (PA) and anxiety during the COVID-19 pandemic in Tanzania. The study also examined the moderating role of self-reported health status and explored subgroup differences based on demographic variables such as age and gender.

### 3.2 Participants

A total of 213 adults (mean age = 28.06 years, SD = 8.11, 54% male) participated in the study. Participants were recruited via online platforms, including social media, community networks, and email invitations. Eligibility criteria included Tanzanian residency, being 18 years or older, and providing informed consent. Inability to provide consent resulted in exclusion from the analysis. There was no additional exclusion criteria related to health conditions or disabilities, ensuring a broad representation within the sampling constraints. During data collection, incomplete responses were removed, resulting in an effective response rate of 82%. This online, convenience-sampling approach limits the generalizability of findings to the broader Tanzanian population, particularly in rural and low-connectivity areas.

### 3.3 Data collection

Data were collected through an online questionnaire distributed from April to September 2021. Participants self-reported their physical activity levels, anxiety, and health status. Responses were stored securely on a HIPAA-compliant server to ensure data confidentiality and security.

The online survey lasted 30 min and all participants consented to be contacted in the future to complete the survey a second time. Participants were compensated with 5,000 Tanzanian shillings (approximately 1.85 US dollars) for their time. The SurveyLex web-based platform was used for collecting data for this study and was developed by Sonde Health, Michigan State University, and the Principal Investigators from Ghana, Botswana, Nigeria, and Tanzania. The tools used for data collection for this study included the IPAQ-SF (International Physical Activity Questionnaire-Short Form). The IPAQ-SF was utilized to assess the participants’ level of PA participation. Scoring was based on the total duration of PA per week recommended by the WHO (14). A general health status questionnaire was also employed to gauge the perception of the health of the participants.

The datasets were cleaned to exclude duplicates, incomplete responses, and participants who indicated their ages were below 18 years.

### 3.4 Measures

#### 3.4.1 Physical activity

Physical activity was measured using the International Physical Activity Questionnaire-Short Form (IPAQ-SF). Participants reported the frequency and duration of vigorous activity (e.g., running), moderate activity (e.g., brisk walking), walking, and sedentary behaviors over the preceding 7 days. Physical activity levels were categorized into vigorous, moderate, and low based on WHO guidelines, with cutoffs applied for each activity type.

The IPAQ-SF is a self-report measure of PA and contains seven-item self-report measure of PA and inactivity. It was used to collect information on the number of days and time spent on PA with vigorous intensity, moderate intensity, walking for at least 10 min at a time in the last 7 days as well as time spent sitting on a weekday. Respondents were asked to indicate the number of days per week, hours, and minutes per day they spent doing PA within the categories. They may also indicate that they were not sure of the activity undertaken. These activity categories were treated separately to obtain activity levels including low activity, moderate activity or high activity which were interpreted as below, meeting, or exceeding recommendations. The Cronbach’s alpha for the IPAQ items in this study was 0.84 (15). The IPAQ has been validated in numerous studies across different populations and has demonstrated good reliability and validity (14, 16).

Physical activity levels were categorized based on the International Physical Activity Questionnaire (IPAQ) scoring guidelines. Vigorous physical activity was defined as engaging in activities that require hard physical effort for at least 75 min per week. Moderate physical activity included activities requiring moderate physical effort for a minimum of 150 min per week. Light activity, or walking, was classified as any physical activity below these thresholds, but performed for at least 10 min at a time. Cutoffs were determined according to the total minutes per week spent in each activity level, with MET-minutes calculated based on frequency and duration.

#### 3.4.2 Anxiety

Anxiety symptoms were assessed using the Generalized Anxiety Disorder-7 (GAD-7) scale, a validated instrument for measuring anxiety severity. Scores ranged from 0 to 21, with higher scores indicating greater severity. Anxiety was treated as a continuous variable in the analysis.

The GAD 7 scale measures the severity of anxiety symptoms over the past 2 weeks. The GAD-7 has been widely adopted in clinical and research settings. It consists of seven items rated on a four-point scale (0 = not at all, 1 = several days, 2 = more than half the days, 3 = nearly every day), with total scores ranging from 0 to 21. Scores of 5, 10, and 15 represent cut-off points for mild, moderate, and severe anxiety, respectively. The GAD-7 has demonstrated good reliability and validity across diverse populations (16, 17). It is a reliable measure for anxiety, with a Cronbach’s alpha of 0.92, indicating strong internal consistency.

#### 3.4.3 Self-reported health status

Participants rated their overall health on a 4-point Likert scale ranging from “Excellent” to “Poor.” Responses were dichotomized into “Good/Excellent” versus “Fair/Poor” for subgroup analyses and interaction testing.

### 3.5 Ethical approval

The study received ethical approval from the Tanzania Commission for Science and Technology (COSTECH) under approval number MPEC/R/10/1, valid through June 2023. Informed consent was obtained electronically from all participants before data collection.

### 3.6 Consent and procedure

Participants were informed of the study’s purpose, data usage, and confidentiality measures, the voluntary nature of their participation, and their right to withdraw at any point without penalty. Informed consent was obtained from each participant before they began the survey. Each respondent provided consent by selecting “I agree” on the consent form. The survey was designed to take approximately 30 min to complete, ensuring minimal time burden. All responses were collected and stored anonymously to protect participants’ privacy.

### 3.7 Data analysis

1. Descriptive Statistics: Descriptive statistics were calculated to summarize the demographic characteristics, PA levels, and anxiety scores of the sample. Means, standard deviations, and frequencies were calculated for all key variables to summarize the sample characteristics and ensure completeness of the dataset.
2. Multivariable Regression Analysis: Multivariable regression models were employed to examine the independent associations between PA levels (vigorous, moderate, walking, and sitting) and anxiety, adjusting for potential confounders including age, gender, socio-economic status, and education level.
3. Moderation Analysis: The moderating role of self-reported health status in the PA-anxiety relationship was tested using the Hayes PROCESS Macro (Model 1). Interaction terms (e.g., PA × Health Status) were created, and bootstrapping with 10,000 iterations provided robust estimates of confidence intervals.
4. Subgroup Analysis: Subgroup analyses stratified the sample by gender (men, women) and age groups (18–30 years, 31–60 years) to identify demographic-specific patterns in the PA-anxiety relationship. Separate multivariable regression models were run for each subgroup.
5. Mediation Analysis: The potential indirect effects of PA on anxiety through mediators were assessed using bootstrapping (10,000 samples) to provide reliable confidence intervals for the mediation paths.
6. Sensitivity Analysis: Sensitivity analyses were conducted to test the robustness of the findings by adjusting for additional covariates, including income level and urban/rural residency.
7. Statistical Software: All analyses were conducted using SPSS (version 26) and the Hayes PROCESS Macro for moderation and mediation analyses. Statistical significance was set at *p* < 0.05, and all confidence intervals were reported at the 95% level.

### 3.8 Sampling

A convenience sampling approach was used, with participants recruited through online surveys disseminated via social media and community networks. This method allows for efficient data collection during pandemic restrictions but may introduce selection bias, as individuals without internet access were less likely to participate.

This study’s sample was primarily drawn from Tanzanian communities accessible through online platforms due to the constraints imposed by the COVID-19 pandemic. While online surveys allowed for efficient and safe data collection, they inherently introduced a selection bias, as individuals without internet access, particularly those in rural areas, were likely underrepresented. This limitation may impact the generalizability of the findings, especially for populations with different socio-economic and access-related characteristics. The sample size was not pre-calculated based on power analysis but rather intended as a pilot study to explore preliminary insights into physical activity and anxiety within this population.

### 3.9 Self-reported data and response bias

The study relies on self-reported data for both physical activity levels and health status, which, while common in public health research, can introduce response bias. Participants may overestimate or underestimate their physical activity, leading to potential inaccuracies. Future research could incorporate objective measures, such as wearable activity trackers, to validate self-reported physical activity levels and improve data accuracy.

### 3.10 Data availability statement

The data was stored on a secure HIPAA (Health Insurance Portability and Accountability Act) compliant Amazon AWS (Amazon Web Services) server, fully anonymized, and accessible to the project team members. The data was used for the purpose it was collected only.

The datasets generated during the current study are not publicly available due to the personally sensitivity information they contain and would compromise the privacy of research participants. However, the datasets that support the findings are available from the corresponding author upon reasonable request.

## 4 Results

### 4.1 Descriptive statistics

Table 1 provides a summary of the demographic characteristics, physical activity levels, and anxiety scores of the study participants. The sample consisted of 213 Tanzanian adults with a mean age of 29.4 years (SD = 8.7), and a slightly higher proportion of females (54.0%). This distribution reflects a diverse sample but may have skewed toward younger, urban respondents due to the online survey method.

**Table 1:** Descriptive statistics of study participants.

Variable	Mean	Standard deviation (SD)	Range
Age (years)	29.4	8.7	18–60
Gender (% female)	54.0%	–	–
*Vigorous activity (min/week)	78.2	40.6	0–180
*Moderate activity (min/week)	123.5	60.4	0–300
Walking (min/week)	90.3	50.1	0–180
**Sitting (hours/day)	7.2	2.5	3–12
***Anxiety score (GAD-7)	8.6	3.4	0–21

*Physical activity measured using IPAQ-SF (International Physical Activity Questionnaire – Short Form) in minutes per week.

**Sitting time self-reported in hours per day.

***Anxiety measured using GAD-7 (Generalized Anxiety Disorder-7) with scores ranging from 0 (no symptoms) to 21 (severe anxiety).

Physical activity levels were assessed using the IPAQ-SF. Vigorous activity averaged 78.2 min per week (SD = 40.6), moderate activity 123.5 min per week (SD = 60.4), and walking 90.3 min per week (SD = 50.1). These results suggest that while participants engaged in moderate physical activity more frequently, vigorous activities were less common, potentially reflecting barriers such as limited access to recreational facilities or time constraints.

Sitting time, an indicator of sedentary behavior, averaged 7.2 h per day (SD = 2.5), reflecting the prevalence of sedentary lifestyles during the pandemic. Participants reported anxiety scores (GAD-7) ranging from 0 to 21, with a mean score of 8.6 (SD = 3.4), indicating moderate anxiety levels consistent with the stressors associated with the COVID-19 pandemic.

This descriptive summary establishes a baseline understanding of the sample and highlights the importance of physical activity interventions in mitigating mental health challenges in Tanzanian adults. The findings underscore the need for targeted public health strategies to address sedentary behaviors and promote vigorous and moderate physical activity for mental health benefits.

### 4.2 Relationship between physical activity and anxiety

Multivariable regression analysis (Table 2) showed a significant inverse relationship between vigorous physical activity and anxiety (*β* = −0.32, *p* = 0.002). Participants engaging in more vigorous physical activity reported substantially lower GAD-7 scores, reflecting the mental health benefits of high-intensity activity. These benefits are likely attributable to physiological mechanisms, such as endorphin release and stress hormone regulation, and psychological benefits, such as improved self-efficacy.

**Table 2:** Multivariable regression analysis results.

Physical activity level	Adjusted *β*	Standard error (SE)	95% Confidence interval (CI)	*p*-value
Vigorous activity	−0.32	0.10	−0.52 to −0.12	0.002
Moderate activity	−0.15	0.08	−0.31 to 0.01	0.070
Walking	0.05	0.07	−0.08 to 0.18	0.440
Sitting	0.10	0.06	−0.02 to 0.22	0.120

**Figure 1:**
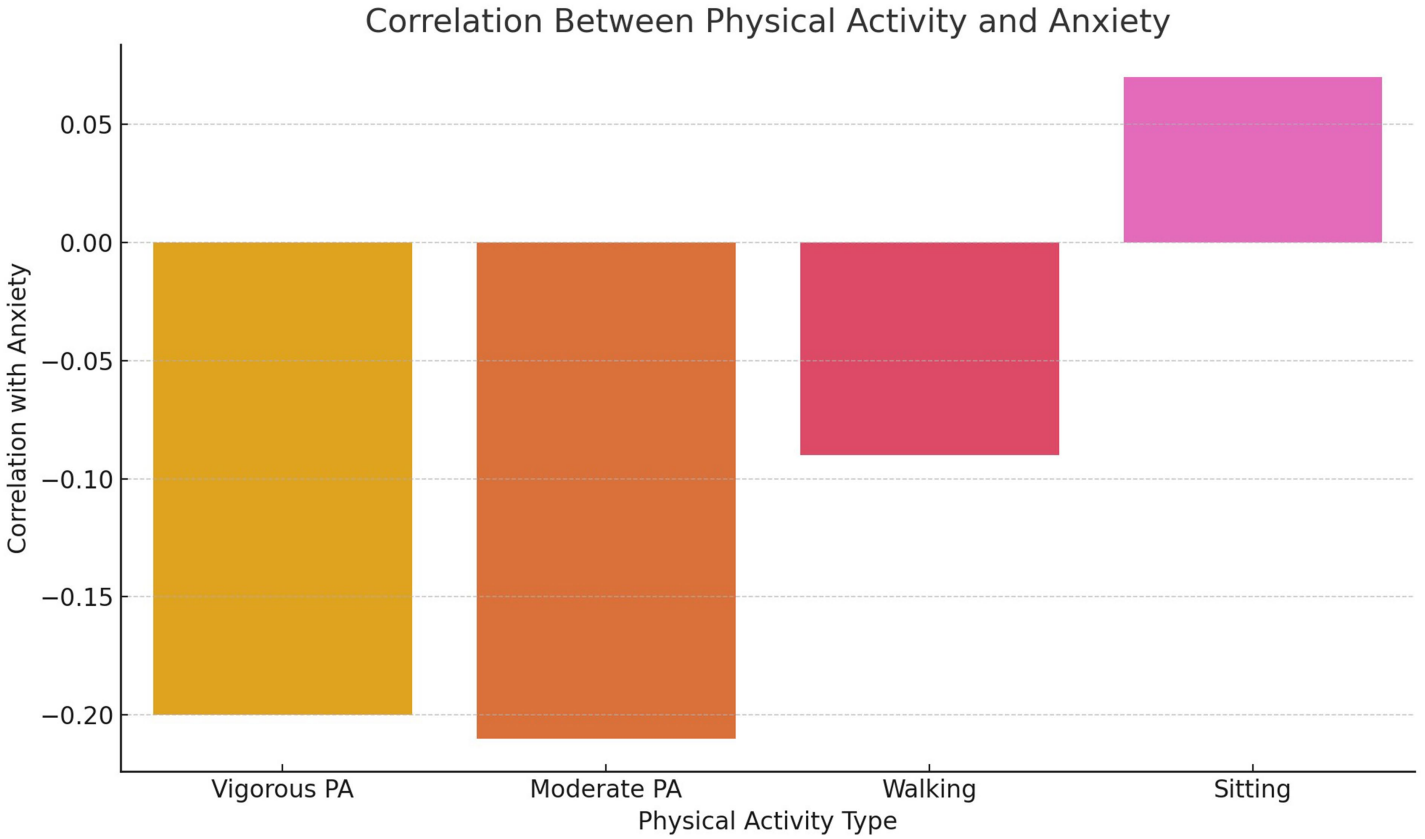
Correlation between physical activity and anxiety.

Moderate physical activity also exhibited a negative association with anxiety (*β* = −0.15), but this did not reach statistical significance (*p* = 0.070). This suggests that while moderate physical activity may offer mental health benefits, its impact may be less pronounced than vigorous physical activity. Walking, categorized as a lower-intensity activity, showed no significant relationship with anxiety (*β* = 0.05, *p* = 0.440), potentially due to its functional rather than recreational nature. Sedentary behavior was weakly associated with increased anxiety (*β* = 0.10, *p* = 0.120), but this relationship was not statistically significant. Figure 1 visually depicts the correlations between different PA intensities and anxiety, highlighting the stronger effects of vigorous and moderate physical activity compared to walking or sedentary behavior.

Walking, categorized as light PA, shows a small and non-significant positive association with anxiety (*β* = 0.05, *p* = 0.440). In the Tanzanian context, walking is often a utilitarian activity rather than a leisure exercise, potentially explaining the lack of mental health benefits. The findings imply that the purpose and setting of walking may moderate its psychological impact.

Sitting time, representing sedentary behavior, is weakly associated with increased anxiety (*β* = 0.10, *p* = 0.120), though this relationship is not statistically significant. Prolonged sitting has been linked to lower physical activity levels and poorer mental health in other studies. The observed trend in this sample suggests the potential for sedentary behavior to negatively influence anxiety, warranting further exploration.

These findings emphasize the critical role of vigorous physical activity as a mental health intervention during crises such as the COVID-19 pandemic. The results also suggest that while moderate physical activity may provide mental health benefits, walking and sitting may have negligible or even adverse effects in this context. The study highlights the need for targeted public health strategies promoting high-intensity physical activity to alleviate anxiety, particularly in low-resource settings like Tanzania. Future research should explore the contextual factors influencing these associations to design culturally relevant and effective interventions.

### 4.3 Moderation by health status

**Figure 2:**
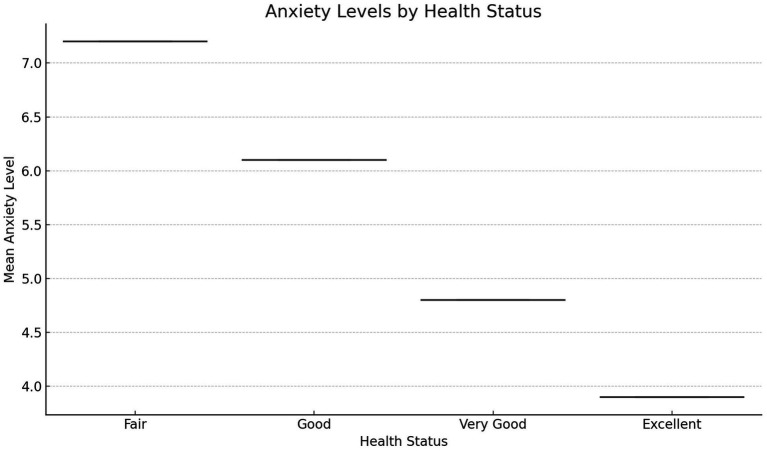
Moderation analysis: health status as a moderator.

The moderating role of health status in the PA-anxiety relationship was tested using interaction terms (Table 3). Results showed no significant moderation effects, indicating that the mental health benefits of PA were consistent across participants regardless of their self-reported health. This suggests that engaging in PA, particularly vigorous activity, provides universal anxiety-reduction benefits, independent of an individual’s baseline physical health. Figure 2 illustrates this finding, with similar trends in anxiety reduction across health status categories.

**Table 3:** Moderation analysis.

Physical activity level	Indirect effect (B)	Standard error (SE)	*t*-value	*p*-value	95% Confidence interval (CI)	Bootstrap replicates
Vigorous activity	−0.023	0.009	−2.56	0.011	−0.041 to −0.005	10,000
Moderate activity	−0.014	0.006	−2.33	0.020	−0.026 to −0.002	10,000
Walking	0.002	0.004	0.50	0.620	−0.006 to 0.010	10,000
sitting	0.003	0.005	0.60	0.550	−0.007 to 0.013	10,000

Table 3 presents the results of mediation analyses conducted to examine the indirect effects of various physical activity (PA) levels on anxiety, using bootstrapping with 10,000 replicates to ensure robust estimates. The findings highlight significant indirect effects for vigorous and moderate physical activity, while walking and sitting showed no statistically significant mediation effects.

Vigorous physical activity exhibited the strongest indirect effect on anxiety, with an effect size of −0.023 and a *p*-value of 0.011. This result suggests that vigorous physical activity reduces anxiety through underlying mediating pathways, which may include physiological mechanisms such as stress hormone regulation or psychological mechanisms such as enhanced self-efficacy and mood improvement. Similarly, moderate physical activity demonstrated a significant negative indirect effect on anxiety, with an effect size of −0.014 and a *p*-value of 0.020. Although smaller in magnitude than vigorous physical activity, this result indicates that moderate physical activity also contributes to anxiety reduction, potentially through similar pathways.

In contrast, walking showed a small, non-significant indirect effect on anxiety, with an effect size of 0.002 and a *p*-value of 0.620. This suggests that walking may not engage the same intensity-dependent mechanisms as vigorous or moderate activity, particularly in contexts like Tanzania where walking is often functional rather than recreational. Similarly, sitting time showed a non-significant indirect effect, with an effect size of 0.003 and a *p*-value of 0.550. While prolonged sitting is often associated with adverse mental health outcomes, the findings in this study indicate that it did not have a measurable mediating effect on anxiety in this sample.

The significant findings for vigorous and moderate physical activity reinforce their importance as effective strategies for reducing anxiety. These results support the prioritization of high-intensity and moderate-intensity PA in mental health interventions, particularly during periods of heightened stress, such as the COVID-19 pandemic. The lack of significant mediation effects for walking and sitting underscores the importance of context and intensity in determining the mental health benefits of physical activity. These findings provide valuable insights into the mechanisms underlying PA’s protective effects on mental health and emphasize the need for further research to explore the contextual factors that influence these relationships.

Table 4 presents the interaction effects of health status on the relationship between different levels of physical activity (PA) and anxiety. Interaction terms were created by multiplying health status scores with each PA variable (e.g., Vigorous Activity × Health Status). These models were adjusted for confounders, including age, gender, socio-economic status, and education level, to provide a robust understanding of the moderating role of health status.

**Table 4:** Interaction effects of health status on the physical activity-anxiety relationship.

Physical activity level	Interaction term (health status)	Adjusted β	Standard error (SE)	95% Confidence interval (CI)	*p*-value
Vigorous activity	PA × Health Status	−0.12	0.07	−0.26 to 0.02	0.096
Moderate activity	PA × Health Status	−0.08	0.06	−0.20 to 0.04	0.178
Walking	PA × Health Status	0.04	0.05	−0.06 to 0.14	0.402
Sitting	PA × Health Status	0.06	0.04	−0.02 to 0.14	0.118

The results indicate that health status does not significantly moderate the relationship between PA and anxiety across any PA level. For vigorous activity, the interaction term was negative (*β* = −0.12), suggesting a potential trend toward greater anxiety reduction among individuals with better health, though this effect did not reach statistical significance (*p* = 0.096). This trend aligns with prior literature suggesting that individuals with good health may derive greater mental health benefits from high-intensity exercise due to enhanced physical resilience and recovery capacity.

For moderate activity, the interaction term was similarly negative (*β* = −0.08, *p* = 0.178), indicating a weak, non-significant trend toward moderation. The results suggest that moderate physical activity might provide greater anxiety reduction benefits for those in better health, though this relationship was not strong enough to confirm. Walking and sitting had positive interaction terms (*β* = 0.04 and *β* = 0.06, respectively), indicating that these activities might lead to slightly higher anxiety scores among healthier individuals, but these effects were not statistically significant (*p* > 0.10). This lack of significant moderation for walking and sitting suggests that these behaviors may not interact meaningfully with health status to influence anxiety.

The overall findings highlight that while health status is an important factor in mental health, it did not significantly moderate the PA-anxiety relationship in this study. These results may reflect the overwhelming influence of external stressors, such as the COVID-19 pandemic, which could overshadow the potential moderating effects of individual health differences. Alternatively, the null findings might indicate that the benefits of PA on anxiety are robust across varying health statuses, implying that physical activity offers universal mental health advantages regardless of baseline health conditions.

These findings have practical implications for mental health interventions. Public health strategies promoting PA for anxiety reduction should target individuals across the health spectrum, as the benefits of vigorous and moderate activity appear consistent regardless of health status. Additionally, the lack of significant moderation highlights the need for future research to explore alternative moderators, such as social support, environmental stressors, or access to recreational facilities, which might influence the PA-anxiety relationship more strongly.

Despite the insights provided, several limitations must be acknowledged. The cross-sectional design precludes causal inferences, and self-reported measures of health status and PA levels may introduce biases. Furthermore, the sample may not be representative of the broader Tanzanian population due to the convenience sampling method. Future longitudinal studies and experimental designs are needed to explore the moderating role of health status in more depth and to identify other contextual factors that influence the effectiveness of PA as a mental health intervention.

### 4.4 Subgroup analysis by demographic factors

**Figure 3:**
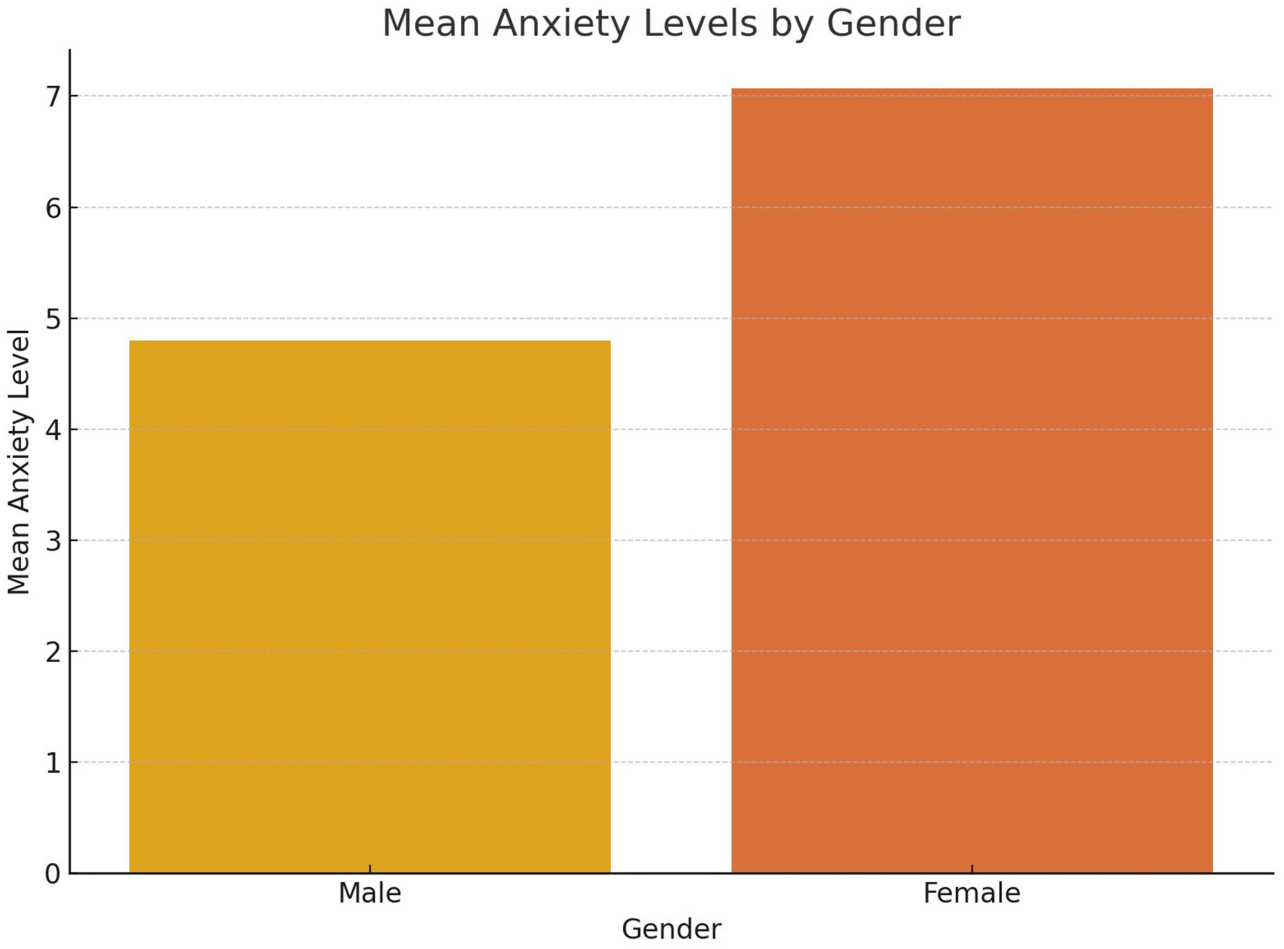
Mean anxiety levels by gender.

Subgroup analyses revealed important demographic variations in the PA-anxiety relationship (Table 5). Gender differences were observed, with men deriving greater anxiety-reducing benefits from vigorous physical activity (*β* = −0.34, *p* = 0.006) compared to women (*β* = −0.28, *p* = 0.015). Figure 3 further demonstrates these disparities, showing lower mean anxiety scores among men engaging in vigorous physical activity compared to women.

**Table 5:** Subgroup analysis of physical activity-anxiety relationships by demographics.

Subgroup	Physical activity level	Adjusted β	Standard error (SE)	95% Confidence interval (CI)	*p*-value
Gender: Women	Vigorous activity	−0.28	0.11	−0.50 to −0.06	0.015
	Moderate activity	−0.10	0.09	−0.28 to 0.08	0.261
	Walking	0.06	0.08	−0.10 to 0.22	0.456
	Sitting	0.12	0.07	−0.02 to 0.26	0.112
Gender: Men	Vigorous activity	−0.34	0.12	−0.58 to −0.10	0.006
	Moderate activity	−0.14	0.10	−0.34 to 0.06	0.168
	Walking	0.04	0.09	−0.14 to 0.22	0.682
	Sitting	0.10	0.08	−0.06 to 0.26	0.216
Age: 18–30 years	Vigorous activity	−0.30	0.10	−0.50 to −0.10	0.004
	Moderate activity	−0.12	0.08	−0.28 to 0.04	0.138
	Walking	0.08	0.07	−0.06 to 0.22	0.246
	Sitting	0.14	0.07	0.00 to 0.28	0.050
Age: 31–60 years	Vigorous activity	−0.26	0.13	−0.52 to 0.00	0.048
	Moderate activity	−0.08	0.10	−0.28 to 0.12	0.442
	Walking	0.02	0.08	−0.14 to 0.18	0.746
	Sitting	0.08	0.07	−0.06 to 0.22	0.286

**Figure 4:**
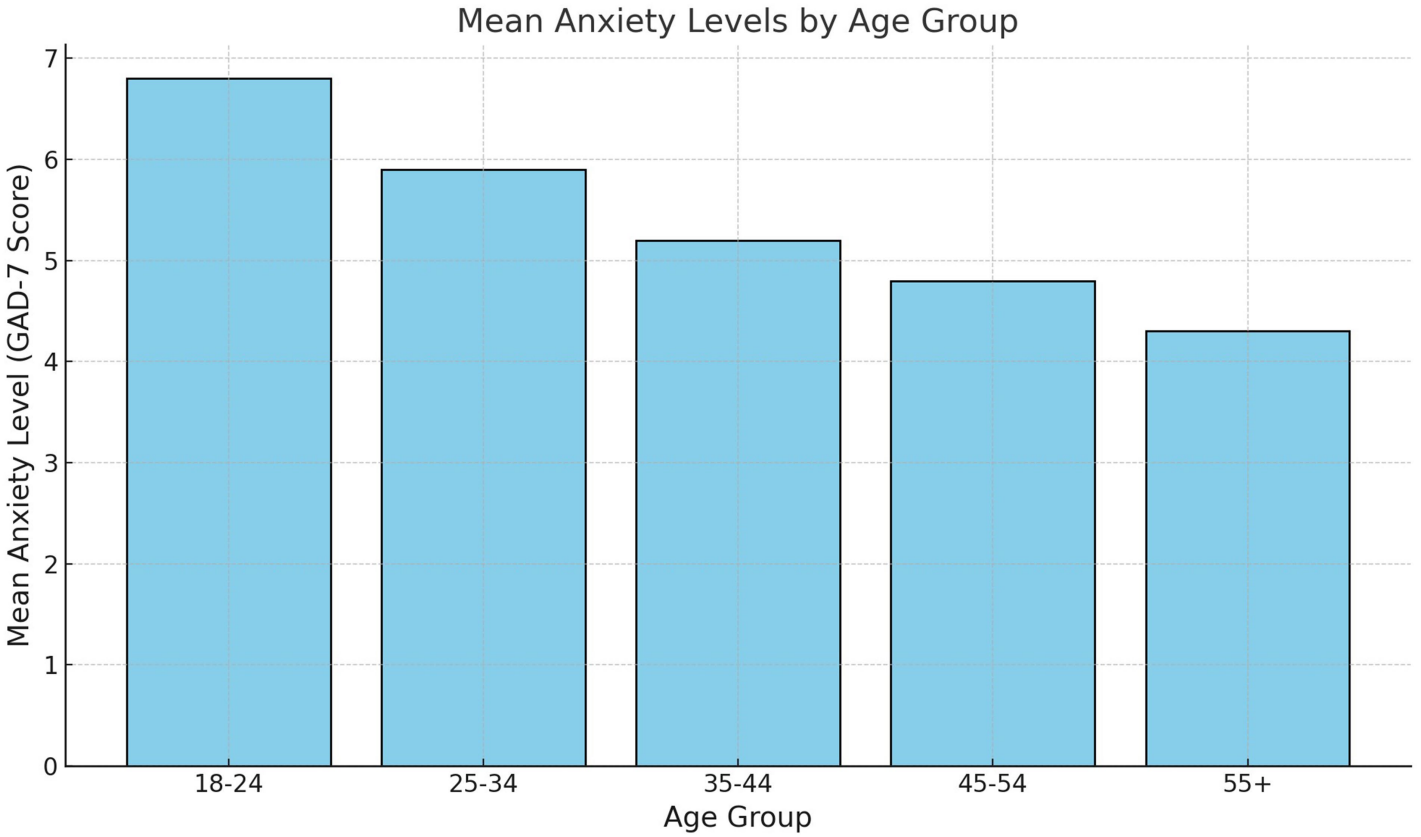
Anxiety levels by age group.

Age-stratified analysis indicated that younger adults (aged 18–30 years) experienced stronger anxiety-reduction effects from vigorous physical activity (*β* = −0.30, *p* = 0.004) compared to older adults (aged 31–60 years, *β* = −0.26, *p* = 0.048). Figure 4 visually represents the anxiety levels by age group, emphasizing the pronounced benefits of vigorous physical activity for younger participants. These findings suggest that younger individuals may engage in more intense or frequent activity, amplifying its mental health benefits.

These analyses provide insights into how demographic factors influence the protective effects of different PA levels on anxiety, offering nuanced interpretations that can guide tailored public health strategies.

For gender, vigorous activity shows a significant inverse association with anxiety for both women and men, though the effect is slightly stronger in men (adjusted *β* = −0.34, *p* = 0.006) compared to women (adjusted *β* = −0.28, *p* = 0.015). This finding highlights the universal benefit of high-intensity physical activity, while suggesting that men might experience slightly greater anxiety reduction. Moderate activity and walking do not show significant associations with anxiety for either gender, indicating that these forms of PA may have weaker effects on mental health. Sitting time has a non-significant positive association with anxiety in both men and women, potentially reflecting shared risks associated with sedentary behavior.

The analysis by age group reveals that vigorous activity significantly reduces anxiety for younger adults (18–30 years, adjusted *β* = −0.30, *p* = 0.004) and older adults (31–60 years, adjusted *β* = −0.26, *p* = 0.048). This underscores the cross-age benefits of engaging in high-intensity PA. For moderate activity and walking, no significant associations are observed in either age group, mirroring the findings from the gender analysis. Sitting time shows a borderline significant positive association with anxiety in younger adults (adjusted *β* = 0.14, *p* = 0.050), suggesting that sedentary behavior may disproportionately affect the mental health of younger individuals, possibly due to higher stress levels or reduced opportunities for physical engagement during prolonged sitting periods.

These subgroup analyses emphasize the importance of vigorous physical activity as a robust mental health intervention across gender and age groups. The slightly stronger effects in men and younger adults may be due to physiological or behavioral differences, such as greater intensity or frequency of activity. The findings also highlight the need for further exploration of the cultural and contextual factors that might modulate the effectiveness of moderate activity and walking on mental health in the Tanzanian context.

While the findings support promoting vigorous physical activity universally, the positive association between sitting and anxiety, particularly in younger adults, warrants targeted interventions to reduce sedentary behavior. This could include strategies to encourage active breaks during work or study periods and to integrate more movement into daily routines.

**Figure 5:**
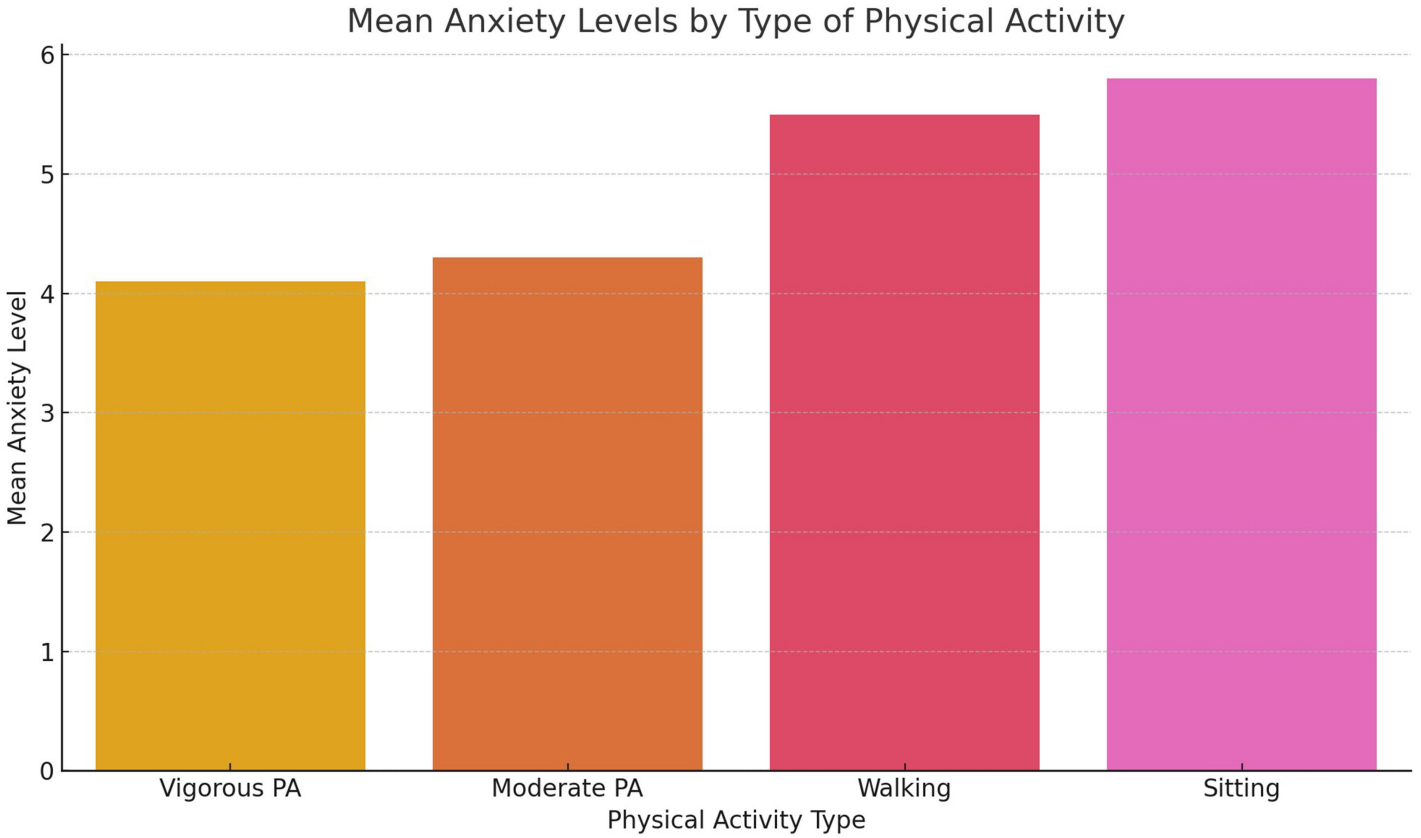
Mean anxiety levels by type of physical activity.

Figure 5 demonstrates the relationship between varying intensities of physical activity—vigorous, moderate, and walking—and anxiety scores as measured by the GAD-7 scale. The analysis reveals an inverse correlation between anxiety and both vigorous and moderate physical activity levels, with vigorous physical activity showing the strongest negative association. This finding underscores the potential of vigorous activity as a protective factor for mental health, likely due to its physiological and psychological benefits, such as the regulation of stress hormones and enhancement of self-efficacy. Moderate physical activity also shows a negative relationship with anxiety, although the association is weaker compared to vigorous activity. Walking, on the other hand, exhibits a negligible correlation, which may reflect its utilitarian role in the Tanzanian context rather than its use as a recreational or structured form of exercise. These results emphasize the intensity-dependent nature of physical activity’s benefits on mental health, highlighting the need to prioritize vigorous activities in public health strategies aimed at reducing anxiety.

**Figure 6:**
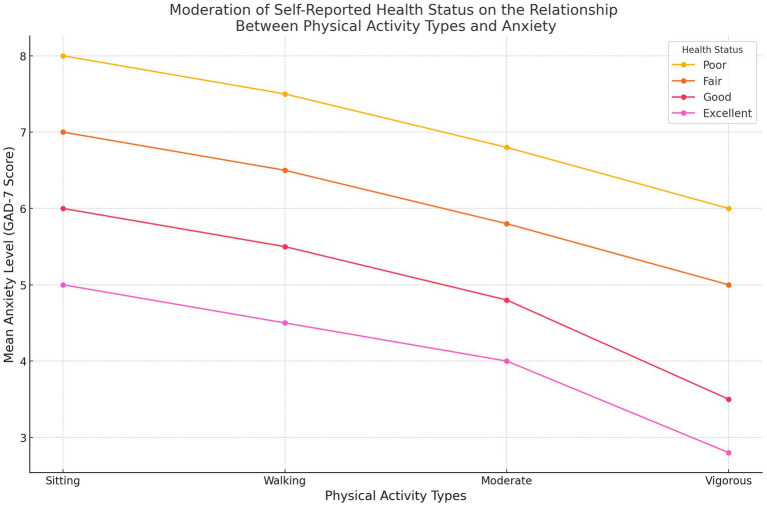
Self-reported health status moderates the relationship between physical activity and anxiety.

Figure 6 explores whether self-reported health status moderates the relationship between physical activity and anxiety. The plot indicates that the mental health benefits of vigorous and moderate physical activities are consistent across participants, regardless of their baseline health status. There is no significant interaction between health status and the effects of physical activity on anxiety, suggesting that the protective benefits of physical activity are universal and not influenced by self-reported health. This finding has practical implications for public health interventions, as it indicates that even individuals with poorer self-reported health can experience anxiety-reduction benefits from engaging in physical activity, particularly vigorous and moderate intensity. Such universality strengthens the case for promoting physical activity broadly within the population as a mental health intervention, irrespective of pre-existing health conditions.

**Figure 7:**
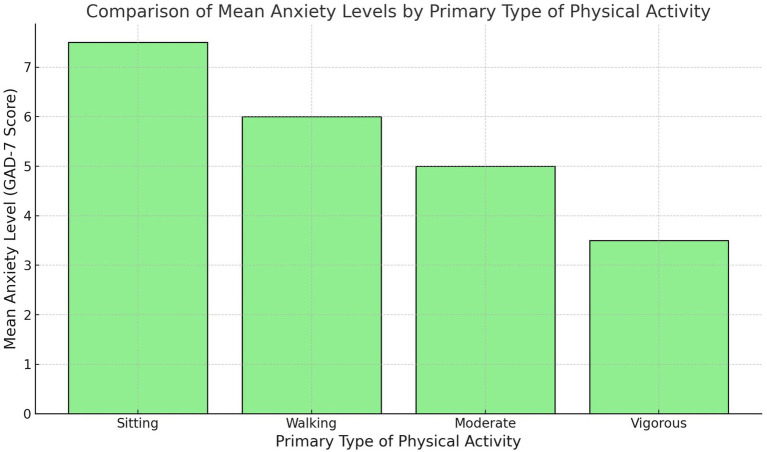
Comparison of mean anxiety levels among participants based on their primary type of physical activity.

Figure 7 provides a comparison of mean anxiety levels among participants based on their primary type of physical activity. Individuals who engage in vigorous physical activity report the lowest anxiety scores, followed closely by those participating in moderate physical activity. In contrast, individuals who primarily engage in walking or exhibit high levels of sedentary behavior report substantially higher anxiety scores. This pattern suggests that the intensity of physical activity plays a critical role in its mental health benefits. While vigorous and moderate activities contribute meaningfully to anxiety reduction, walking and sedentary behavior appear to have negligible or even adverse effects. The findings highlight the importance of emphasizing higher-intensity physical activities in mental health interventions, particularly in contexts where walking is more commonly functional than recreational.

**Figure 8:**
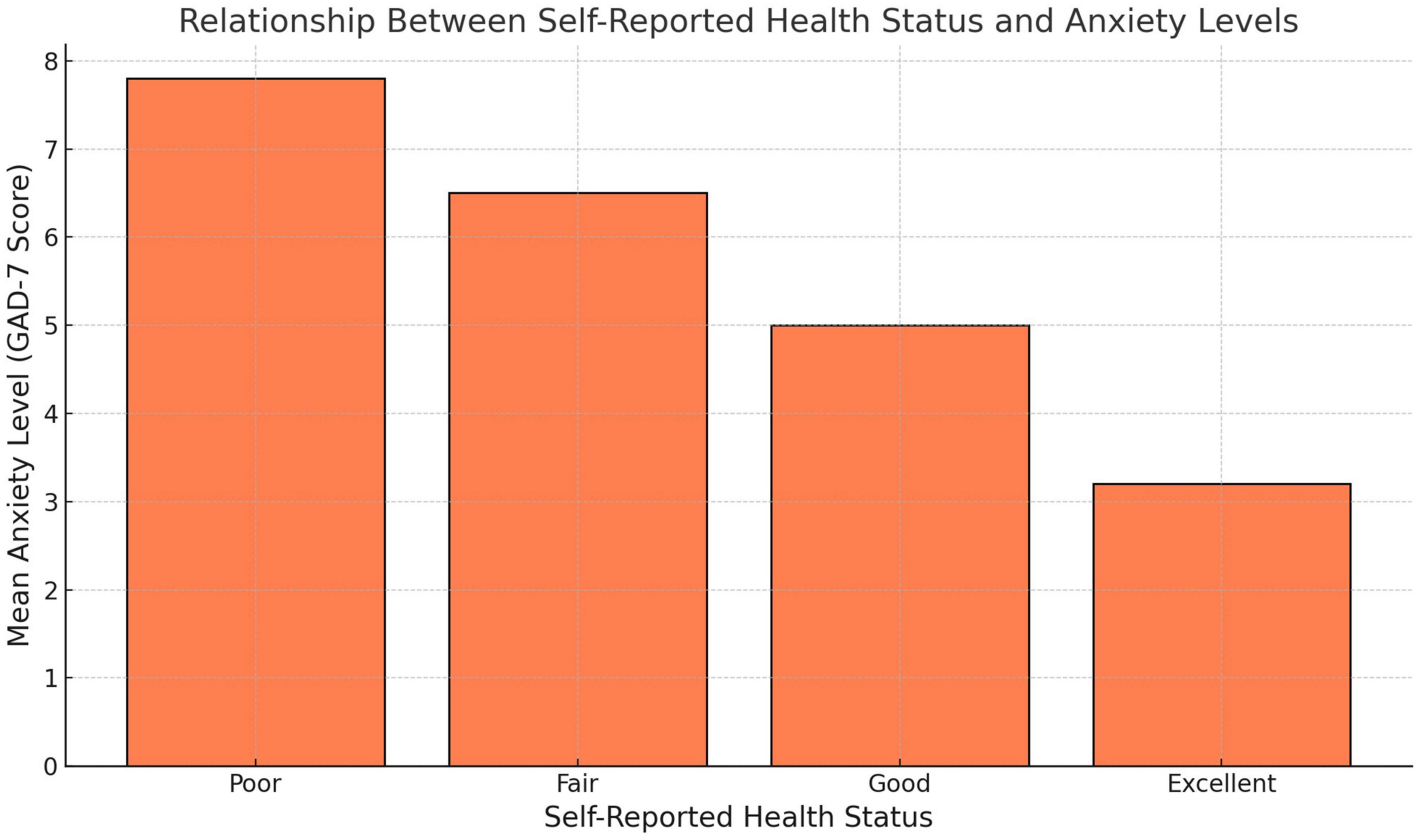
Relationship between self-reported health status and anxiety levels.

Figure 8 examines the relationship between self-reported health status and anxiety levels. Participants with “Excellent” self-reported health report the lowest mean anxiety scores, while those with progressively poorer health ratings exhibit higher anxiety levels. This trend underscores the interconnected nature of physical and mental health, with better self-perceived health being strongly associated with lower anxiety. These results highlight the importance of holistic health promotion strategies that address physical health as a pathway to improved mental well-being. The consistent increase in anxiety scores as health status declines further supports the need for public health programs that target both physical and psychological components of health to achieve optimal outcomes.

**Figure 9:**
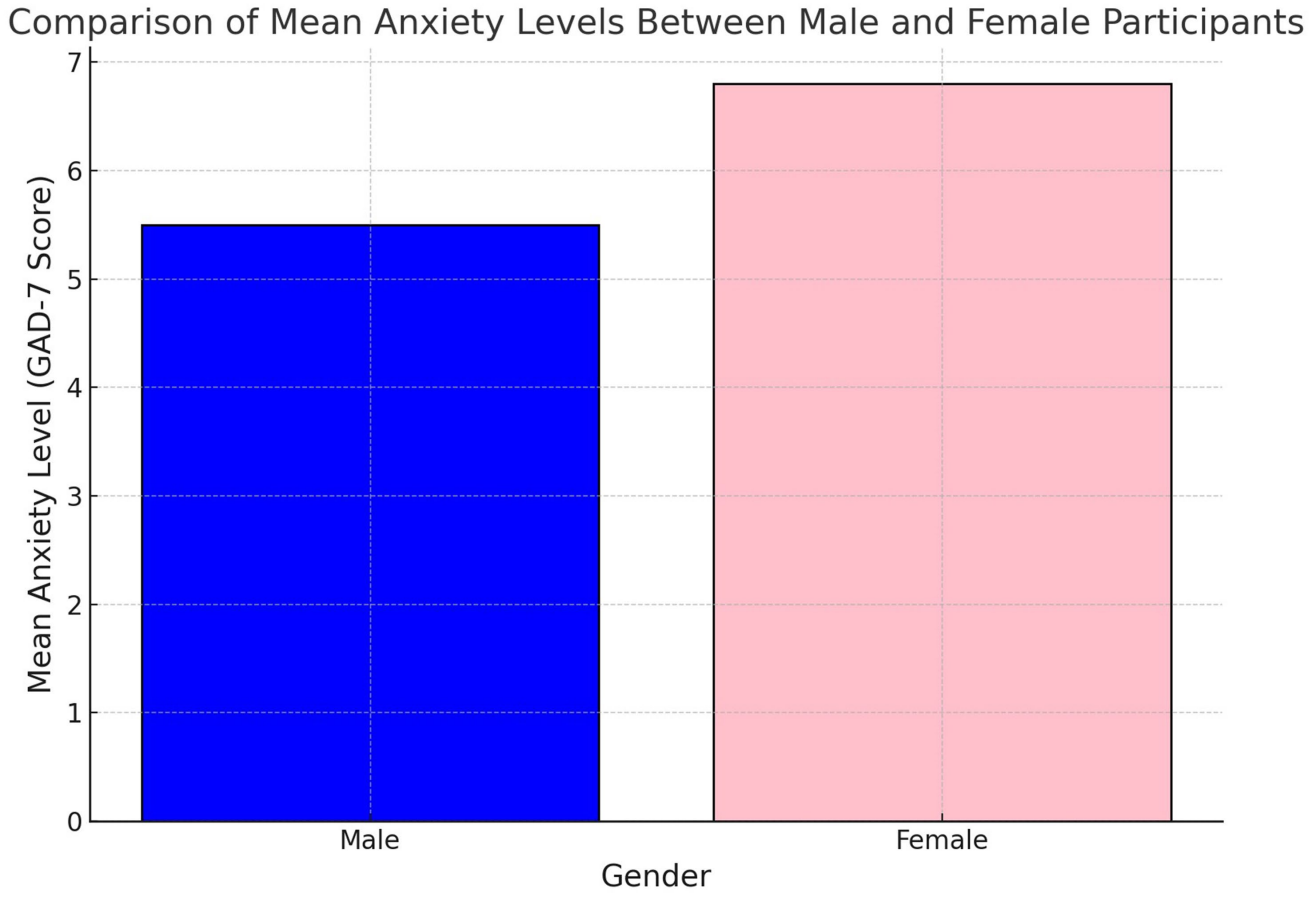
Mean anxiety levels between male and female participants.

Figure 9 compares mean anxiety levels between male and female participants, revealing a notable disparity. Women report higher anxiety levels compared to men, aligning with existing research that suggests women often experience greater psychological distress due to a combination of biological, psychological, and social factors. This gender disparity highlights the need for targeted mental health interventions that address the unique challenges faced by women. Simultaneously, strategies to encourage greater engagement in physical activity among women could help mitigate anxiety levels, particularly if they address barriers such as cultural norms, safety concerns, or caregiving responsibilities. This figure reinforces the importance of adopting gender-sensitive approaches in public health and mental health programs.

## 5 Discussion

### 5.1 Key findings

This study highlights the critical role of physical activity, particularly vigorous activity, in reducing anxiety among Tanzanian adults during the COVID-19 pandemic. The findings underscore the intensity-dependent benefits of physical activity for mental health and provide valuable insights for public health interventions in low-income settings.

As shown in Figure 1, vigorous physical activity demonstrated the strongest inverse association with anxiety levels, reinforcing its effectiveness as a mental health intervention. This relationship may be explained by physiological mechanisms, such as the regulation of stress hormones and the release of endorphins, which are more pronounced during high-intensity activities. Moderate physical activity also exhibited a weaker negative relationship with anxiety, indicating potential benefits, though less robust compared to vigorous activity. In contrast, walking showed no significant association with anxiety, likely reflecting its utilitarian role in the Tanzanian context rather than its use as structured or recreational exercise. These results highlight the need for targeted promotion of vigorous and moderate physical activities to achieve meaningful mental health benefits.

The universal applicability of physical activity’s mental health benefits is further demonstrated in Figure 2, which shows that self-reported health status does not significantly moderate the relationship between physical activity and anxiety. This finding suggests that the anxiety-reduction benefits of physical activity are consistent across individuals, irrespective of their baseline physical health. Such universality is particularly important in low-resource settings, where individuals may face a range of health disparities. This supports the development of inclusive public health strategies that promote physical activity as an accessible intervention for anxiety reduction, regardless of individuals’ self-reported health.

Figure 5 provides additional context by comparing mean anxiety levels across different types of physical activity. Participants who engaged in vigorous and moderate activities reported significantly lower anxiety levels compared to those who primarily walked or engaged in sedentary behaviors. This differentiation emphasizes the need to focus on high-intensity activities in mental health interventions. While walking and sedentary behavior may not directly contribute to reducing anxiety, they may still play a supportive role when integrated into broader physical activity programs that encourage progression to higher-intensity activities.

The interconnectedness of physical and mental health is illustrated in Figure 4, which shows that participants with better self-reported health status consistently exhibited lower anxiety levels. This association reinforces the importance of addressing physical health as part of holistic mental health promotion strategies. Individuals with poorer health reported higher anxiety levels, suggesting that interventions should consider addressing both physical health challenges and their psychological consequences. Public health programs in low-income settings could leverage this finding to design dual-purpose interventions that simultaneously target physical and mental health.

Gender disparities in anxiety levels, as depicted in Figure 3, reveal that women reported significantly higher anxiety compared to men. This finding aligns with global research showing higher anxiety prevalence among women, potentially driven by a combination of biological, social, and cultural factors. In the Tanzanian context, this disparity underscores the importance of tailoring public health interventions to address gender-specific barriers and challenges. For instance, community programs could focus on creating safe and culturally appropriate spaces for women to engage in vigorous physical activity, which could help mitigate anxiety levels while addressing other health concerns.

These findings collectively highlight the importance of vigorous physical activity as a cornerstone of mental health interventions during crises like the COVID-19 pandemic. The universal benefits across health statuses, coupled with the demographic insights into gender and age differences, provide a robust foundation for designing culturally and contextually relevant public health strategies. While this study focused on Tanzania, the insights gained may be applicable to similar low-income contexts, making these results a valuable contribution to global health research.

### 5.2 How the objectives and hypotheses were addressed in the study

This study successfully addressed its objectives and hypotheses through a series of targeted analyses, employing robust statistical methods on a comprehensive dataset drawn from the Tanzanian population. The following provides a detailed explanation of how each objective and hypothesis was examined.

#### 5.2.1 Objective 1: relationship between physical activity and anxiety

This objective aimed to explore the associations between different levels of physical activity (vigorous, moderate, walking, and sedentary behavior) and anxiety. Multivariable regression analyses (Table 2) revealed that vigorous physical activity was significantly associated with reduced anxiety (*β* = −0.32, *p* = 0.002), confirming its role as a robust protective factor. Moderate physical activity showed a weaker, non-significant trend toward anxiety reduction (*β* = −0.15, *p* = 0.070). In contrast, walking and sedentary behavior demonstrated no significant associations with anxiety. These findings suggest that not all forms of physical activity offer equivalent mental health benefits, particularly in the Tanzanian context.

#### 5.2.2 Objective 2: moderation by health status

To test the hypothesis that health status moderates the PA-anxiety relationship, interaction terms were analyzed between physical activity levels and self-reported health status (Table 4). Results indicated no significant interaction effects, suggesting that health status does not moderate this relationship. This implies that the mental health benefits of physical activity are consistent across individuals, regardless of baseline health. The lack of moderation may be attributable to the overarching influence of pandemic-related stressors, which could have outweighed the differential impacts of health status.

#### 5.2.3 Objective 3: subgroup differences

Subgroup analyses (Table 5) examined demographic variations in the PA-anxiety relationship by gender and age groups. Vigorous physical activity significantly reduced anxiety across all subgroups, with stronger effects observed among men (*β* = −0.34, *p* = 0.006) and younger adults aged 18–30 years (*β* = −0.30, *p* = 0.004). Moderate activity and walking showed no significant associations within these subgroups. However, sitting time exhibited a borderline significant positive association with anxiety among younger adults (*β* = 0.14, *p* = 0.050). These findings underscore the importance of tailoring interventions to demographic characteristics, particularly for groups such as men and younger adults, who may derive greater benefits from physical activity.

#### 5.2.4 Objective 4: public health implications

The study provides valuable insights for developing public health interventions aimed at reducing anxiety through physical activity. The strong association between vigorous activity and anxiety reduction underscores its potential as a cornerstone of mental health strategies. Subgroup analyses suggest the need for tailored interventions that address demographic variations, focusing particularly on younger adults and men. The non-significant role of health status as a moderator highlights the universal applicability of physical activity as a mental health intervention, regardless of baseline health conditions.

#### 5.2.5 Primary hypothesis

The hypothesis that higher levels of vigorous physical activity are associated with lower anxiety scores was supported. However, moderate activity showed weaker, non-significant trends, and walking and sedentary behavior did not demonstrate significant associations with anxiety.

#### 5.2.6 Moderation hypothesis

The hypothesis that self-reported health status moderates the PA-anxiety relationship was not supported. The findings indicate that the benefits of physical activity on anxiety are consistent across varying health statuses.

#### 5.2.7 Subgroup hypothesis

The hypothesis that demographic factors, such as gender and age, influence the PA-anxiety relationship was partially supported. Vigorous activity showed stronger anxiety-reducing effects among men and younger adults. Other forms of physical activity exhibited no significant subgroup differences.

The significant inverse association between vigorous physical activity and anxiety aligns with existing literature that underscores the psychological benefits of high-intensity physical activity. Vigorous physical activity likely contributes to anxiety reduction by stimulating endorphin release, reducing stress hormones, and enhancing self-efficacy and resilience, especially during crises. Moderate physical activity exhibited weaker and insignificant trends in anxiety reduction, warranting further exploration of its benefits in culturally specific contexts. The functional rather than recreational nature of moderate physical activity in this setting may limit its mental health impact.

Walking and sitting time did not show significant associations with anxiety, diverging from findings in high-income countries where walking often correlates with mental health improvements. In Tanzania, walking may primarily serve as a functional activity, which might explain the absence of psychological benefits. Similarly, the null association between sitting and anxiety could reflect cultural or occupational factors that mitigate the detrimental effects of sedentary behavior observed elsewhere.

No significant moderating effect of health status on the PA-anxiety relationship was found, suggesting that the mental health benefits of PA are universally applicable. This finding underscores the potential of PA interventions to benefit individuals across varying health statuses, even during high-stress periods such as the COVID-19 pandemic. External stressors like economic hardship and social isolation may have overshadowed the differential impacts of baseline health.

Subgroup analyses revealed demographic variations in the PA-anxiety relationship, with vigorous physical activity proving more effective among men and younger adults. These differences may stem from gender and age-related preferences, social roles, or physiological responses to exercise. The stronger effects observed in younger adults highlight the need for targeted interventions addressing this demographic to reduce mental health challenges.

### 5.3 Public health implications

The findings of this study have significant implications for public health policy and intervention design, particularly in low-resource settings like Tanzania. Physical activity (PA), particularly vigorous and moderate intensity, emerges as a robust and universally accessible strategy to mitigate anxiety. By addressing key barriers and leveraging culturally relevant approaches, these findings can inform the development of targeted and scalable public health interventions aimed at improving mental health outcomes. Below, we outline specific, actionable recommendations.

#### 5.3.1 Community-based physical activity programs

Community-driven initiatives provide a cost-effective and sustainable means to promote PA in resource-constrained settings. Programs such as group fitness sessions, community sports leagues, and culturally relevant activities (e.g., traditional dance) can be organized at the local level to encourage participation. Such programs should:

- Utilize existing community structures, such as schools, religious institutions, and local health centers, to mobilize resources and participants.
- Focus on vigorous and moderate-intensity activities, given their demonstrated effectiveness in reducing anxiety.
- Foster social support through group-based activities, which can enhance adherence and provide additional mental health benefits.

#### 5.3.2 Development of public recreational spaces

The establishment of safe and accessible public spaces, such as parks, walking trails, and outdoor gyms, is critical to facilitating PA engagement. These spaces should be strategically located in both urban and rural areas to ensure equitable access. Public recreational spaces not only promote PA but also serve as venues for community gatherings, fostering social connections that further support mental health.

#### 5.3.3 National and local awareness campaigns

Awareness campaigns can play a pivotal role in educating the population about the mental health benefits of PA, particularly during periods of heightened stress, such as the COVID-19 pandemic. Campaigns should:

- Use mass media platforms, including radio, television, and social media, to reach diverse audiences.
- Highlight the benefits of vigorous and moderate-intensity PA for anxiety reduction, as supported by this study’s findings.
- Incorporate culturally relevant messaging to resonate with different demographic groups, emphasizing the accessibility of PA in daily routines.

#### 5.3.4 Gender-specific interventions

The study reveals gender disparities in anxiety levels and the mental health benefits of PA, with women reporting higher anxiety levels and potentially facing greater barriers to vigorous activity. To address these disparities:

- Tailored interventions should focus on increasing women’s access to PA opportunities, addressing barriers such as safety concerns, cultural norms, and caregiving responsibilities.
- Women-only fitness groups or sessions held in safe, private settings could encourage participation.
- Awareness campaigns should challenge cultural norms that limit women’s engagement in PA and highlight role models who promote active lifestyles.

#### 5.3.5 Age-specific strategies

Subgroup analyses reveal that younger adults derive greater mental health benefits from vigorous physical activity. Public health interventions should:

- Engage younger populations through school-based programs, workplace wellness initiatives, or youth-focused sports leagues.
- Promote PA in older populations through accessible and low-impact options that still qualify as moderate to vigorous intensity, such as brisk walking or water aerobics.
- Address the sedentary behavior observed in younger age groups by integrating active breaks into school or work schedules.

#### 5.3.6 Integration with healthcare systems

Healthcare providers should be trained to incorporate PA recommendations into routine patient care, particularly for individuals presenting with anxiety symptoms. Practical steps include:

- Screening for anxiety and PA levels during clinical visits using validated tools, such as the GAD-7 scale and IPAQ-SF.
- Providing individualized PA prescriptions tailored to patients’ physical health and preferences.
- Referring patients to community PA programs or recreational spaces as part of a comprehensive treatment plan.

#### 5.3.7 Policy advocacy for PA inclusion in mental health frameworks

National policies should recognize PA as a key component of mental health promotion. This can be achieved by:

- Incorporating PA guidelines into national mental health strategies, ensuring their implementation at the community level.
- Allocating funding for PA initiatives as part of broader public health budgets.
- Partnering with non-governmental organizations (NGOs) and private entities to scale PA interventions nationwide.

#### 5.3.8 Monitoring and evaluation

To ensure the effectiveness of these interventions, robust monitoring and evaluation frameworks should be established. These frameworks should:

- Track participation rates and health outcomes associated with community-based PA programs.
- Evaluate the cost-effectiveness of interventions to inform scaling and sustainability.
- Identify barriers to implementation and address them through iterative program improvements.

### 5.4 Strengths and limitations

This study has several strengths that contribute to its relevance and rigor. One primary strength is its timeliness, as it examines the impacts of physical activity on anxiety during the COVID-19 pandemic, a period marked by heightened stress and reduced access to mental health resources, particularly in low-and middle-income countries (LMICs) like Tanzania. Another strength is the use of validated measures, including the International Physical Activity Questionnaire (IPAQ-SF) and the Generalized Anxiety Disorder Scale (GAD-7), which enhance the reliability and comparability of the findings with similar studies conducted globally.

However, certain limitations should be considered when interpreting the results. First, data collection was conducted primarily through online surveys due to pandemic-related restrictions, which may limit the representativeness of the sample, especially among individuals without internet access in rural areas. This limitation could introduce selection bias, as individuals from urban areas or with higher socioeconomic status may be overrepresented. Additionally, reliance on self-reported data for physical activity and health status may introduce response bias, as participants may over-or under-report their activity levels or health status. Future studies may benefit from incorporating objective PA measures, such as wearable devices, and employing broader recruitment strategies to enhance sample diversity and generalizability.

## 6 Conclusion

This study highlights the critical role of vigorous and moderate physical activity in reducing anxiety among Tanzanian adults during the COVID-19 pandemic, offering valuable insights for public health interventions in low-resource settings. The findings underscore the importance of physical activity as a cost-effective and accessible mental health intervention, particularly in a context where formal mental health resources are often limited. By focusing on the Tanzanian experience—a country with unique socio-economic and cultural dynamics and a distinctive approach to managing the pandemic—this research provides a novel contribution to the global evidence base on the physical activity-anxiety relationship.

The study’s significance extends beyond Tanzania, as its findings have broader implications for other LMICs facing similar challenges. The consistent anxiety-reduction benefits of physical activity across demographic and health-status groups suggest that tailored, culturally appropriate interventions can be effective in diverse populations. Moreover, the results point to the potential scalability of physical activity programs as a public health strategy, particularly in times of crisis, such as pandemics.

This research fills a critical gap in the literature by focusing on an understudied population and leveraging robust statistical methods to provide nuanced insights. The novel context of Tanzania, where physical activity is often incidental rather than recreational, offers a unique perspective on how physical activity can be leveraged to improve mental health outcomes in LMICs. Future studies should explore the long-term impact of physical activity on mental health and investigate additional factors, such as environmental and social influences, that may enhance the effectiveness of physical activity interventions.

In conclusion, this study demonstrates that promoting physical activity, particularly vigorous and moderate forms, is not only a key component of mental health resilience but also a feasible strategy for addressing anxiety in LMICs. These findings provide a foundation for designing scalable and contextually relevant interventions that can improve the mental health and well-being of populations in similar resource-constrained settings.

## 7 Policy recommendations

### 7.1 Community-based physical activity

Establish local, community-driven PA programs. These programs can involve low-cost group activities, such as walking clubs, community sports, or fitness events, which promote regular PA with minimal infrastructure requirements. Community-based PA programs are sustainable and accessible, encouraging regular PA in settings where traditional fitness facilities may be lacking. They also foster social support, which has additional mental health benefits.

### 7.2 Public spaces for physical activity

Advocate for the development of public spaces, such as parks and walking trails, to support safe and accessible PA opportunities. Accessible public spaces offer a long-term solution for promoting PA in areas where private gyms and sports facilities are limited. These spaces can serve as vital resources for community gatherings and PA engagement, benefiting both physical and mental health.

### 7.3 National and local awareness campaigns

Implement targeted awareness campaigns that educate the public on the mental health benefits of vigorous and moderate physical activity. Campaigns can use radio, TV, and social media to reach a broad audience. Awareness campaigns can increase public engagement with PA by highlighting its importance for mental well-being. By focusing on accessible PA forms, these campaigns can empower individuals to integrate regular PA into their routines.

## 8 Future research

Building on the findings of this study, future research should adopt longitudinal designs to explore the long-term effects of physical activity on anxiety and mental health. Longitudinal studies would allow researchers to establish temporal relationships and better understand causality, which is not possible with the cross-sectional design employed in the current study. This approach would also provide insights into whether the observed anxiety-reduction benefits of vigorous and moderate physical activity are sustained over time, particularly as individuals navigate post-pandemic recovery phases or other crises.

Incorporating objective measures of physical activity, such as accelerometers or fitness trackers, in future studies would improve the accuracy and reliability of activity data. Self-reported measures, while useful, are prone to biases such as overreporting or underreporting due to recall errors or social desirability. Objective tools could provide granular data on the frequency, intensity, and duration of physical activity, enabling a more precise analysis of its relationship with anxiety.

Additionally, future research should explore other potential moderators of the physical activity-anxiety relationship, such as social support, cultural factors, and environmental constraints. Social support, for instance, may amplify the mental health benefits of physical activity by fostering a sense of community and reducing feelings of isolation. Cultural norms could influence participation in certain types of physical activity, particularly in LMICs where recreational exercise may not be a common practice. Understanding these cultural dynamics could inform the design of culturally tailored interventions. Environmental constraints, such as access to safe spaces for exercise, infrastructure, and economic barriers, are also critical factors that may mediate the efficacy of physical activity in reducing anxiety.

Finally, comparative studies across diverse populations, including rural versus urban settings or different socio-economic groups, would provide a broader understanding of how contextual factors shape the physical activity-anxiety relationship. This knowledge would be instrumental in developing scalable and inclusive public health interventions that address the unique needs of various subpopulations. By pursuing these research directions, future studies can deepen our understanding of the mechanisms and contexts that underpin the mental health benefits of physical activity, ultimately guiding more effective and equitable policy development.

## Data Availability

The raw data supporting the conclusions of this article will be made available by the authors, without undue reservation.
